# Acromion and Distal Clavicle Grafts for Arthroscopic Glenoid Reconstruction

**DOI:** 10.3390/jcm12124035

**Published:** 2023-06-13

**Authors:** Jeffrey A. Zhang, Patrick Lam, Julia Beretov, George A. C. Murrell

**Affiliations:** 1 UNSW Faculty of Medicine, Kensington, Sydney, NSW 2033, Australia; 2 Orthopedic Research Institute, St. George Hospital, Kogarah, Sydney, NSW 2217, Australia

**Keywords:** bony Bankart, glenoid defect, glenoid reconstruction, bone graft, biomechanics, recurrent dislocation, suture anchor, screw-free, technique, arthroscopic

## Abstract

Background: We intended to determine if an acromion or distal clavicle bone graft could restore large glenoid defects using two novel, screw-free graft fixation techniques. Methods: Twenty-four sawbone shoulder models were divided into four groups (n = 6 per group) according to fixation technique and bone graft: (1) modified buckle-down technique with clavicle graft, (2) modified buckle-down technique with acromion graft, (3) cross-link technique with acromion graft, (4) cross-link technique with clavicle graft. Testing was performed sequentially in (1) intact models, (2) after creation of a 30% by-width glenoid defect and (3) after repair. The shoulder joint was translated anteriorly, and glenohumeral contact pressures and load were measured to quantify the biomechanical stability. Results: Maximum contact pressures were restored to 42–56% of intact glenoid using acromion and clavicle grafts with novel fixation techniques. Acromion grafts attained higher maximum contact pressures than clavicle grafts in all groups. Peak translational forces increased by 171–368% after all repairs. Conclusions: This controlled laboratory study on sawbone models found that both the acromion and distal clavicle are suitable autologous bone graft options for treating large anterior glenoid defects, having appropriate dimensions and contours for reconstructing the glenoid arc. The modified buckle-down and cross-link techniques are two graft fixation techniques that restore stability to the shoulder joint upon repairing a large glenoid defect and are advantageous in being screw-free and simple to execute.

## 1. Introduction

A number of authors have recommended using bone grafting procedures to restore the glenoid shape when treating large glenoid defects of approximately 6 mm to 8 mm bone loss, or 20–30% of the glenoid width [1,2,3,4,5]. Several bone grafting techniques for glenoid reconstruction have been described. The Bristow and Latarjet procedures transfer the ipsilateral coracoid process and its attached conjoint tendon onto the anterior glenoid [6,7]. Free bone grafts including the iliac crest, distal tibial allografts, femoral condyle and clavicle have also been documented [8,9,10,11,12,13]. Moroder et al. [14] found similar clinical and radiological outcomes between the Latarjet procedure and an iliac crest graft transfer, suggesting that shoulder stability can be restored with free bone grafts alone without requiring additional tendinous or ligamentous attachments.

Anatomical studies into autologous bone grafts found both the acromion and distal clavicle graft have comparable dimensions to the coracoid process and can restore similar-sized glenoid defects when compared to the Latarjet procedure [15,16,17]. However, biomechanical evidence supporting an acromion or distal clavicle graft is limited, and no studies have tested the integrity of these grafts in repairs.

Bicortical screws are one method for graft fixation [7,18]. However, screw-related complications in glenoid reconstruction procedures range from 7% to 48% [19,20]. Malpositioned screws risk neurovascular injury, non-reunion and graft fracture, while loose, painful, broken or migrated screws are a common reason for revision surgery [21,22]. Boileau et al. [23] developed an arthroscopic screw-free Latarjet procedure using cortical buttons and sutures and achieved a 91% union rate, similar to the performance of screw fixation techniques. Although this technique avoided complications reported with screw fixation at 14-month follow-up, the surgery is complicated, requiring longer operative times compared to a free bone graft transfer and presenting a steeper learning curve [24,25]. One previous biomechanical study has tested a novel, screw-free fixation technique using an osteotomised glenoid fragment as the graft. However, the applicability of this screw-free fixation technique on free bone grafts was not assessed [26].

We were interested in determining if an acromion or distal clavicle bone graft could restore the glenoid articular surface area after the creation of a large anterior glenoid defect. The second aim was to develop two screw-free fixation techniques and investigate if they were biomechanically sound for repairing large anterior glenoid defects and restoring shoulder stability.

## 2. Materials and Methods

The study used sawbone models to evaluate two graft fixation techniques and two free bone grafts for the reconstruction of an anterior glenoid. Twenty-four sawbone models of the left scapula (SKU:1050-15, Scapula with Vise Attachment, Foam Cortical, Left, Sawbones, Pacific Research Company, Vashon, WA, USA) were obtained and provided the control and defect states of the glenoid and the acromion grafts. Twelve sawbone models of the clavicle (SKU:1020-20, Clavicle, Solid Foam, Left, Sawbones, Pacific Research Company, Vashon, WA, USA) were obtained for the clavicle grafts. The 24 scapula models were divided into four groups of six scapulae according to the repair technique and bone graft: (1) modified buckle-down technique with clavicle graft, (2) modified buckle-down technique with acromion graft, (3) cross-link technique with acromion graft, (4) cross-link technique with clavicle graft. A total of four humerus sawbone models (SKU: 1013 Humerus, Foam Cortical Shell, Left, Medium Sawbones, Pacific Research Company, Vashon, WA, USA) were used for testing, allowing a fresh humerus for each of the five repair groups.

### 2.1. Defect Creation

The glenoid dimensions of the intact scapula were measured using a digital calliper. The borders of the glenoid were marked at the 12, 3, 6 and 9 o’clock positions. The 12 o’clock position was defined at the supraglenoid tubercle. The remaining 3, 6 and 9 o’clock positions on the left-sided scapula model corresponded with the posterior, inferior and anterior borders of the glenoid, respectively. The centre of the glenoid was defined as being equidistant from the 3, 6 and 9 o’clock positions. The creation of the glenoid defect was adapted from a previous biomechanical study described by Smith et al. [26]. The glenoid defect was created with a motorised saw at a 60-degree angle to the glenoid surface.

### 2.2. Graft Preparation

**Figure 1:**
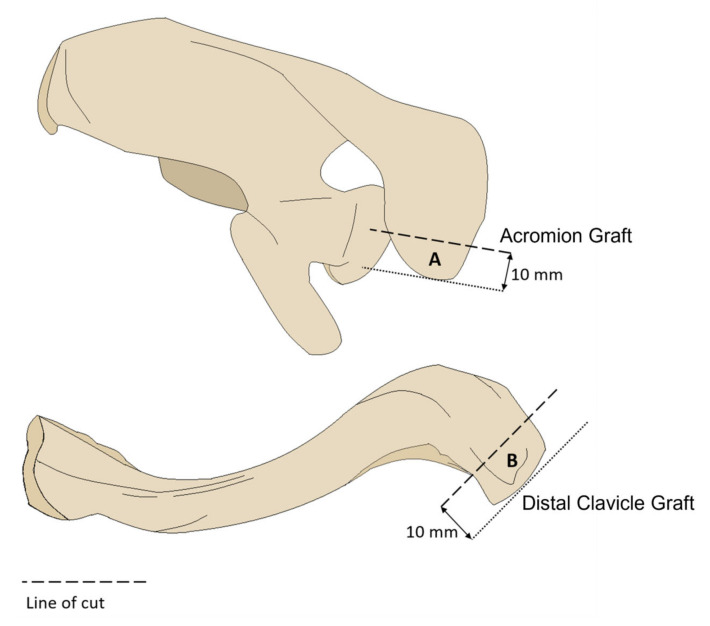
Sawbone models of scapula and clavicle. (A) Superior view of left scapula sawbone model. Acromion graft obtained from a single vertical cut 10 mm from the anterior edge of the acromion process. (B) Superior view of left clavicle sawbone model. Clavicle graft obtained from a single vertical cut 10 mm from the anterolateral edge of the distal clavicle.

Free bone grafts were obtained from the acromion and clavicle. The acromion was marked 10 mm from its anterior edge, and a single vertical cut using a handsaw was made to obtain the acromion graft (Figure 1). The acromion graft was oriented such that its inferior surface formed the articular surface of the glenoid after the repair. The clavicle was marked 10 mm from its distal edge anterolaterally. A single cut perpendicular to the clavicle’s long axis was made to obtain the clavicle graft (Figure 1). The clavicle graft was similarly orientated such that its inferior surface formed the articular surface of the glenoid after the repair.

In all bone grafts, two bone tunnels were hand-drilled parallel to the articular surface. These bone tunnels were 5 mm medial to the articular surface and spaced approximately 10 mm apart. For repairs with the modified buckle-down technique, a 1.5 mm drill bit was used, consistent with the description by Smith et al. [26]. For repairs with the cross-link technique, a 1.8 mm drill bit was used to facilitate the different orientation of the Endobuttons which required a larger bone tunnel.

### 2.3. Fixation Technique

**Figure 2:**
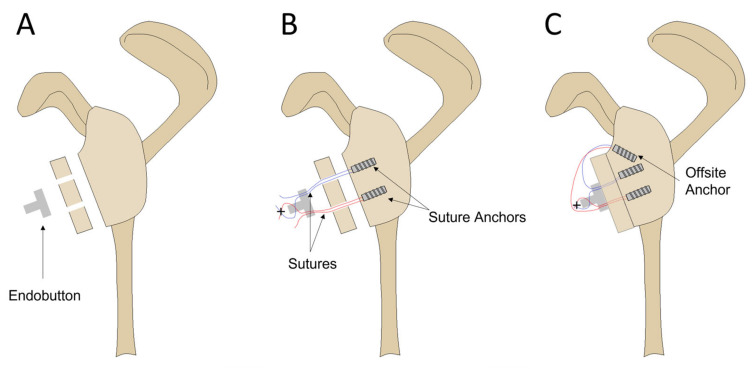
The modified buckle-down repair technique. (**A**) A 30% by-width anterior glenoid defect was created on the scapula. Two 1.5 mm diameter bone tunnels were drilled through the bone graft spaced approximately 10 mm apart. The Endobutton fixation device oriented with notched eyelet facing outwards. (**B**) Two suture anchors were inserted at the defect site with sutures passed through the graft and Endobutton. The medial suture strands were passed in alternating directions through the notched eyelet, and five alternating half-hitch knots were tied at the location marked with a cross. (**C**) Outside sutures were pulled, causing the graft to retract to the defect site. An offsite anchor was placed superiorly to maintain tension and provide bone-to-bone compression.

Two screw-free graft fixation techniques for the glenoid defect were investigated: a modified buckle-down technique [26] (Figure 2) and the cross-link technique, which is an original technique developed by the authors. Both techniques used two suture anchors at the defect site with a third offsite anchor that provided tension in the sutures to achieve bone-to-bone compression of the graft to the glenoid defect. For each repair, the articular surface of the free bone graft was placed flush against the glenoid defect. A K-wire was passed through the predrilled bone tunnels of the free bone graft to mark the anchor locations on the glenoid defect. The location for the offsite anchor was positioned approximately 10 mm superiorly from the superior border of the glenoid defect. The anchor sites were punched with a 3.5 mm sharp-tipped obturator, and SwiveLock single-loaded suture anchors (3.5 mm SwiveLock PEEK, 3.5 mm × 15.8 mm, AR-2325 anchors with #2 Fiberwire; Arthrex, FL, USA) were inserted into the defect site.

### 2.4. Modified Buckle-Down Technique

**Figure 3:**
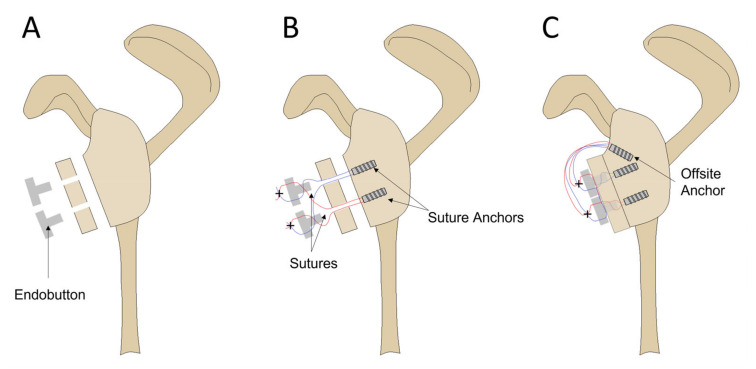
The cross-link repair technique. (**A**) A 30% by-width anterior glenoid defect was created on the scapula. Two 1.8 mm diameter bone tunnels were drilled through the bone graft spaced approximately 10 mm apart. Two Endobutton fixation devices oriented with notched eyelet facing inwards. (**B**) Two suture anchors were inserted at the defect site with sutures passed through the graft and Endobutton in the shown arrangement. Five alternating half-hitch knots were tied at the locations marked with a cross. (**C**) Suture strands were pulled to retract the graft onto the defect site. A Knotpusher was used to impact the eyelet of the Endobuttons into the bone tunnels of the graft. An offsite anchor was placed superiorly to maintain tension and provide bone-to-bone compression.

The suture strands of the superior and inferior anchors were passed through the respective holes in the free bone graft and Endobutton (Figure 3) (Round Endobutton, S2 3/4 Suture Loop; Smith & Nephew, Andover, MA, USA). Of the four free suture strands emerging from the Endobutton, the medial two sutures were passed through the central notched eyelet in alternating directions and then tied with five alternating half-hitch knots. This was a modification to the technique described by Smith et al. [26] as the alternating directions created a self-locking system once additional tension was applied. The two remaining free sutures were loaded into a third anchor and inserted into the superior offsite hole at the glenoid neck.

### 2.5. Cross-Link Technique

The sutures of the superior and inferior anchors were first passed through their respective holes in the free bone graft (Figure 3). Two Endobuttons were used with the notched eyelet facing inwards. The lateral suture of the superior anchor and the medial suture of the inferior anchor were passed in alternating directions through the central notched eyelet of the superior Endobutton and then through its corresponding Endobutton hole, creating a self-locking system similar to that described previously. The two remaining sutures were passed in a similar fashion through the inferior Endobutton. Once the free bone graft was in position, tension was applied to the free sutures, and the eyelet of each Endobutton was impacted into its corresponding bone tunnel using a Knotpusher (Mitek, 6C3 Knotpusher). Due to the size of the eyelet, this required bone tunnels to be drilled with a 1.8 mm drill bit. Five alternating half-hitch knots were tied at each Endobutton. All four free suture strands were loaded into a third anchor and inserted into the superior offsite hole at the glenoid neck.

### 2.6. Testing Protocol

**Figure 4:**
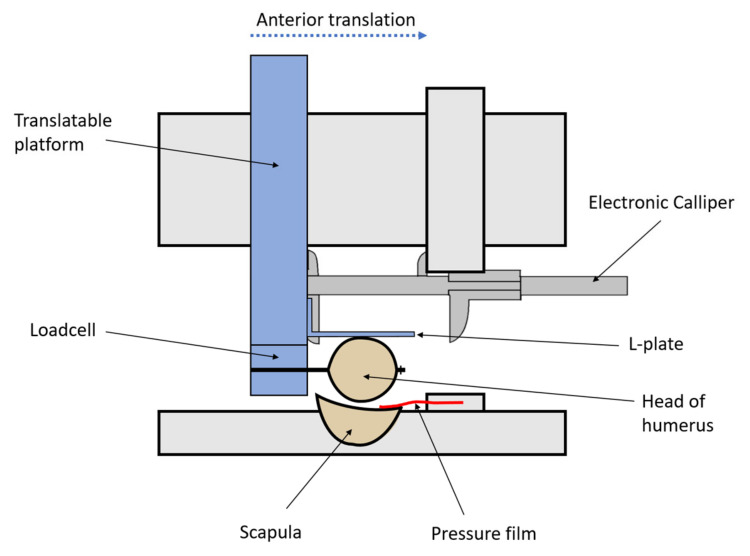
Schematic diagram of the apparatus set up for testing as viewed from above. The L-plate was fastened to the translatable platform, preventing lateral displacement of the humeral head. The scapula was clamped in a multidirectional vice mounted on the table. As anterior translation occurred, the electronic calliper automatically measured the displacement.

Testing was performed under three sequential conditions for each of the five groups: (1) control glenohumeral joint with no osseous defect of the anterior glenoid rim, (2) after the creation of a 30% by-width defect of the anterior glenoid rim, (3) after the repair technique (Figure 4). In each humerus sawbone model, an 8 mm hole was drilled through the humeral head (perpendicular to the bicipital groove). This allowed fixation of a loadcell (HFG-45 loadcell, Transducer Techniques, Temecula CA, USA) to the humerus. The loadcell was mounted on a translatable platform capable of controlled movement in the anterior–posterior plane with displacement measured by an electronic calliper.

Glenohumeral adduction was fixed by aligning the humeral shaft parallel to the defect line on the glenoid such that any anterior–posterior force would be perpendicular to the defect line. An L-plate was fastened against the lateral border of the humeral head to maintain this position between tests and directed the anterior translational force linearly against the glenoid rim by preventing lateral displacement of the humeral head.

The measurements of the contact pressure film (FlexiForce B201-H, Tekscan, MA USA) were electronically recorded as the humerus was translated anteriorly. The repair time from drilling of the glenoid to the insertion of the final anchor was measured as a secondary outcome.

### 2.7. Intraclass Correlation Coefficients and Statistical Analysis

The reliability of the testing apparatus was assessed by testing 30 glenoids in their intact state (Table 1). Two-way random-effects intraclass correlation coefficients (ICCs) with 95% confidence intervals were calculated using IBM SPSS, version 26. All measurements had an ICC value greater than 0.8 which represented excellent reliability. Outcomes measured included the load at 2 mm displacement, 4 mm displacement and peak translational force, as well as contact pressures at 2 mm displacement, 4 mm displacement and maximum contact pressure. Within each group, analysis was performed using one-way ANOVA with repeated measures. Comparisons between groups were performed using one-way ANOVA on ranks (Kruskal–Wallis test) with Dunn’s correction.

## 3. Results

### 3.1. Glenoid and Graft Dimensions

The glenoid dimensions (mean ± SD) of the 24 intact scapula sawbone models had an anterior–posterior width of 30.3 ± 0.2 mm. A total of 12 acromion grafts and 12 clavicle grafts with dimensions shown in Table 2 were used in repairs.

### 3.2. Secondary Outcome Measurements

The modified buckle-down repair technique took an average of 5 min (range, 4.5 to 6 min) to complete. The cross-link technique required an average of 10.5 min (range, 9 to 11.2 min) to complete.

As the joint was dislocated through testing, the mechanism for failure for all repairs was noted to be secondary to mechanical suture stretching. The sutures from the inferior anchors that passed through the inferior bone tunnel of the free bone grafts demonstrated greater mechanical stretching in all repair specimens.

### 3.3. Contact Pressures

**Figure 5:**
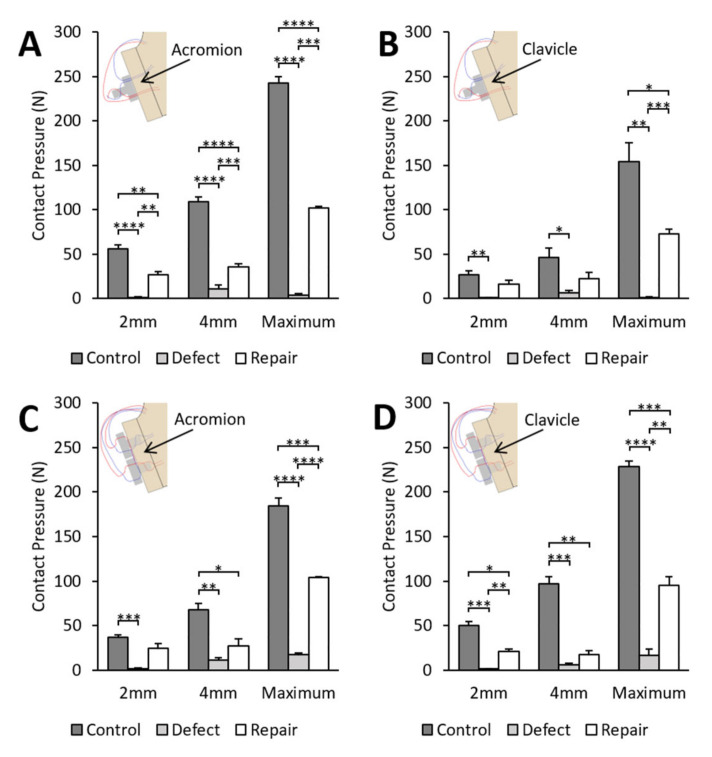
Contact pressure at 2 mm, 4 mm and maximum contact pressure at dislocation between the humeral head and glenoid for four scenarios. In each group, testing was performed for control glenoid, 30% by-width defect glenoid and after repair with (**A**) modified buckle-down technique with acromion, (**B**) modified buckle-down technique with clavicle, (**C**) cross-link technique with acromion or (**D**) cross-link technique with clavicle. Values are expressed as mean ± standard error of the mean with n = 6 in each group. * *p* < 0.05, ** *p* < 0.01, *** *p* < 0.001, **** *p* < 0.0001. Comparisons within groups were performed using one-way ANOVA with repeated measures.

In all groups, the maximum contact pressure obtained at dislocation and at displacements of 2 and 4 mm decreased by 84–99% after the creation of a 30% by-width glenoid defect compared to the control glenoid. After repair, the maximum contact pressure increased significantly in all groups compared to the defect glenoid, achieving 42–56% of the control glenoid value (Figure 5).

**Figure 6:**
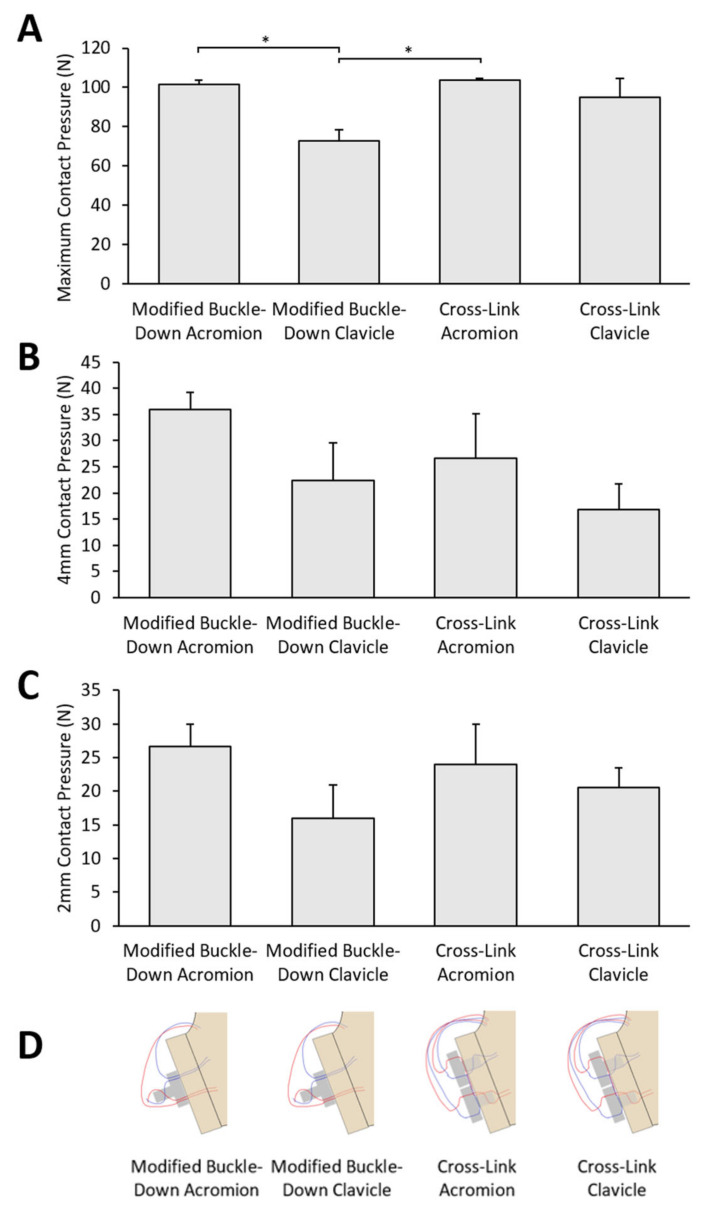
Contact pressures after repair for the four groups. (**A**) Maximum contact pressure. The maximum contact pressure of the modified buckle-down technique with clavicle was significantly lower than the modified buckle-down technique with acromion and cross-link technique with acromion. (**B**) Contact pressure at 4 mm. (**C**) Contact pressure at 2 mm. (**D**) Diagrams of corresponding repair. * *p* < 0.05. Comparisons between groups were performed using one-way ANOVA on ranks (Kruskal–Wallis test) with Dunn’s correction.

The maximum contact pressure (mean ± SEM) of the modified buckle-down technique with clavicle was the lowest (73 ± 6 N), which was 47% of the control. This was significantly lower (*p* < 0.05) than the modified buckle-down technique with acromion (102 ± 2 N) and cross-link technique with acromion (104 ± 1 N) (Figure 6).

At 4 mm displacement, clavicle graft repairs attained 36–39% lower contact pressures than acromion grafts. At 2 mm, clavicle graft repairs attained 14–40% lower contact pressures than acromion grafts (Figure 6).

### 3.4. Peak Translational Force and Load

**Figure 7:**
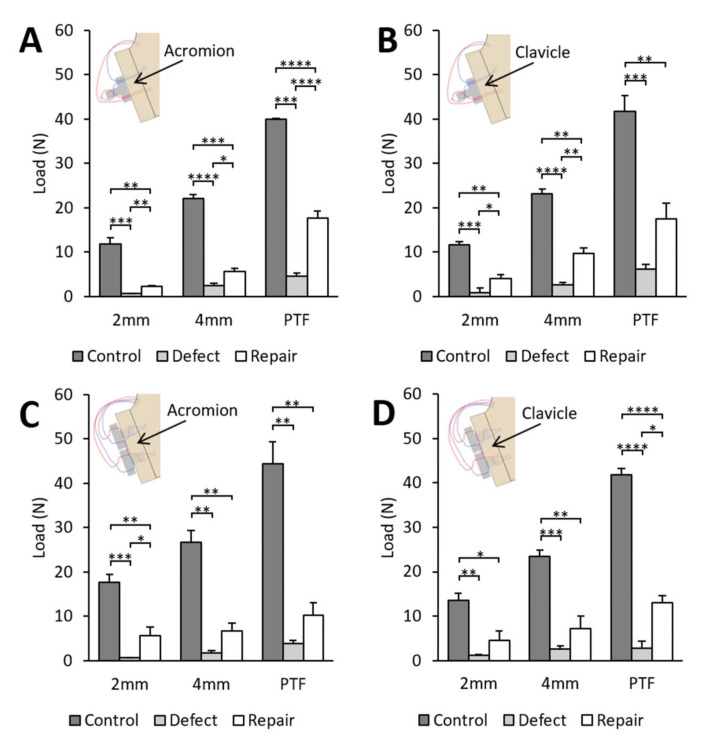
Load at 2 mm, 4 mm and peak translational force at dislocation between the humeral head and glenoid for four scenarios. In each group, testing was performed for control glenoid, 30% by-width defect glenoid and after repair with (**A**) modified buckle-down technique with acromion, (**B**) modified buckle-down technique with clavicle, (**C**) cross-link technique with acromion or (**D**) cross-link technique with clavicle. PTF: peak translational force. Values are expressed as mean ± SEM with n = 6 in each group. * *p* < 0.05, ** *p* < 0.01, *** *p* < 0.001, **** *p* < 0.0001. Comparisons within groups were performed using one-way ANOVA with repeated measures.

In all repair groups, the load at 2 mm, load at 4 mm and peak translational force obtained at dislocation decreased by 85–97% after the creation of a 30% by-width glenoid defect compared to the control glenoid. After repair, the peak translational force increased by 171–368% compared to the defect glenoid, achieving between 23 and 44% of the control glenoid value (Figure 7).

**Figure 8:**
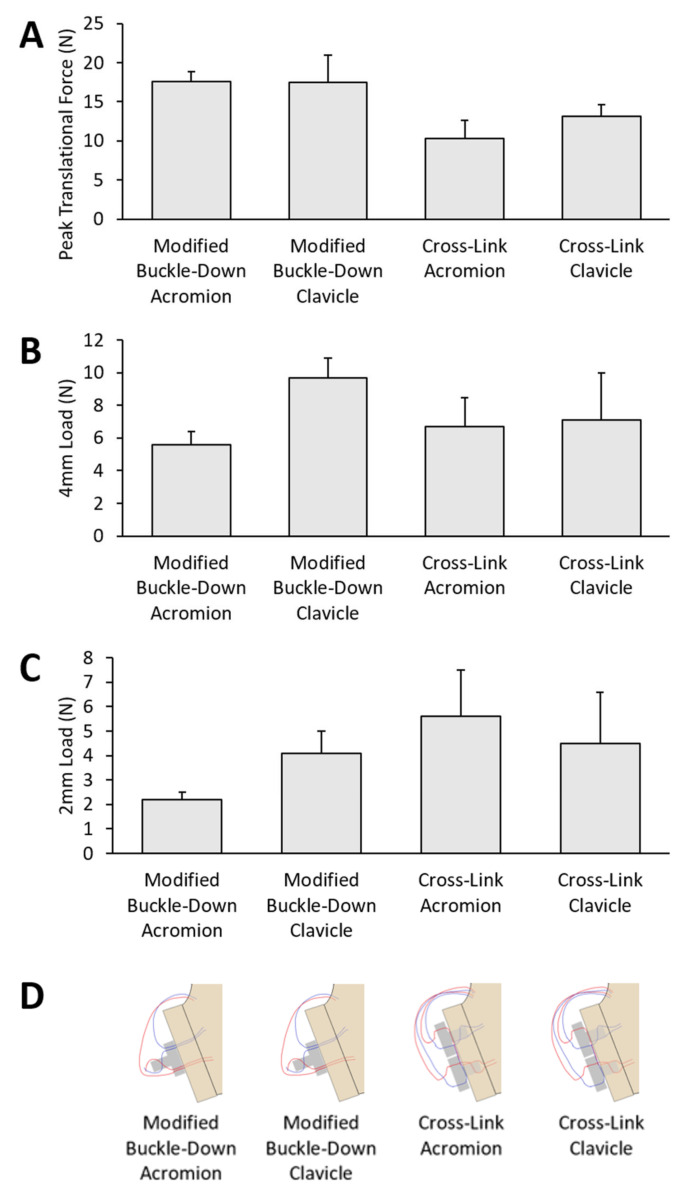
The load after repair for the four groups. (**A**) Peak translational force. (**B**) Load at 4 mm. (**C**) Load at 2 mm. (**D**) Diagrams of corresponding repair. Comparisons between groups were performed using one-way ANOVA on ranks (Kruskal–Wallis test) with Dunn’s correction.

The peak translational force (mean ± standard error of mean) of the modified buckle-down technique with acromion was the highest (18 ± 2 N), which was 44% of the control (40 ± 0.3 N). The cross-link technique with clavicle restored the lowest peak translational force (10 ± 3 N), which was 23% of the control (44 ± 5 N) (Figure 8).

At 4 mm displacement, the modified buckle-down technique with clavicle restored the highest load (10 ± 1 N), which was 42% of control (23 ± 1 N). At 2 mm displacement, the cross-link technique with acromion restored the highest load (6 ± 2 N), which was 32% of the control (18 ± 2 N) (Figure 8).

## 4. Discussion

This study used sawbone models to investigate an acromion and distal clavicle bone graft in reconstructing large glenoid defects. Two novel screw-free graft fixation techniques were used for repairs. Both the acromion and clavicle grafts were large enough to reconstruct a 30% by-width glenoid defect, and maximum contact pressures were restored to 42–56% of intact glenoid values. The modified buckle-down technique and cross-link technique both restored joint stability equally well, with repairs improving peak translational forces by 171–368% compared to the defect glenoids.

No prior studies have biomechanically tested an acromion graft for glenoid reconstruction. Only one previous biomechanical study examined a distal clavicle graft in repairing large glenoid defects. Petersen et al. [27] found the distal clavicle autograft was a suitable option for treating glenoid bone loss as it restored similar contact pressures in the shoulder compared to a coracoid graft used in the Latarjet procedure. This study noted that repairs using clavicle grafts had lower maximum contact pressures than repairs using acromion grafts. The lower contact pressures observed in the clavicle graft repairs may be explained by the curved clavicular articular surface, closely resembling the natural glenoid contour. In comparison, the acromion articular surface was flat, and the anterior humeral head translation likely concentrated the force on a smaller area on the pressure sensor, leading to higher maximum contact pressures. In this study, the curved articular surfaces and lower contact pressures of using a clavicle graft for repairing glenoids may potentially reduce postoperative degenerative arthritis in the glenohumeral joint.

Harvesting a bone graft from the acromion or distal clavicle allows a single surgical operating field and may reduce donor site morbidity seen with iliac crest graft harvests [12,28]. This study obtained an acromion graft using a single vertical osteotomy 10 mm from the anterior acromion edge instead. An anterior acromion graft is simple to harvest, and its smaller size may minimise disruption to local anatomy, especially the attached deltoid muscle, which may simplify the procedure and potentially reduce postoperative complications.

Distal clavicle graft harvests for glenoid reconstruction have been performed clinically by Tokish et al. [11]. The authors found the distal clavicle advantageous in providing an osteochondral component that can mimic glenoid cartilage. Anatomical studies have also identified the distal clavicle can restore 22% to 44% of the glenoid surface area, enough to reconstruct large-sized glenoid defects [15,29]. This study similarly found that a 10 mm distal clavicle graft could restore a 30% by-width glenoid defect.

The buckle-down technique was first described by Smith et al. [26] as a screw-free graft fixation alternative. The authors repaired eleven ovine glenoids with large anterior defects using the osteotomised glenoid fragment as a graft. Shoulder stability calculated from mean force–displacement curves was observed to increase by a magnitude of 2 to 3 times following repair. Smith et al. [26] noted the primary mechanism of failure to be the loosening of the fixation technique secondary to mechanical stretching of the sutures. This study observed a comparable increase of 2 to 4 times in peak translational forces upon repair of defect glenoids. We observed a similar mechanical suture stretching phenomenon in all repairs, with sutures observed to pull away from the implanted anchors particularly as the joint neared dislocation.

Taverna et al. [30] previously described a double Endobutton fixation technique with two trans-glenoid tunnels to reduce the rotational instability of the graft. The cross-link technique was developed to share the load between two Endobuttons and minimise graft rotation while avoiding the need to drill through the entire glenoid, thus simplifying the surgical technique. A case–control comparative study by Barret et al. also noted that additional buttons, such as a four-button fixation technique, did not have any anatomical or clinical advantages compared to a double-button fixation technique. Additional buttons were detrimental as they made the procedure more complex and increased operating time [31]. In our study, we noted no graft rotation in the cross-link technique or modified buckle-down technique. Both techniques performed similarly well in restoring load and contact pressures, although the modified buckle-down technique was simpler to execute, taking on average half the time to repair (5 min) compared to the cross-link technique (10.5 min).

A strength of this study was using standardised sawbone models allowing for a reproducible 30% by-width glenoid defect and consistent bone graft shape and size. Both fixation techniques in this study avoided using screws, reducing the risk of intraoperative neurovascular injury and postoperative hardware complications [32,33,34]. The size of grafts and instruments used could also potentially cater towards an arthroscopic approach.

There are several limitations to this study. The sawbone models simplify glenohumeral stability mechanisms by testing in the absence of other dynamic stabilisers. Shoulder stability was tested in a single neutral arm position across all groups, which is unlikely to represent commonly seen clinical dislocation presentations of the arm in an abducted and externally rotated position [2]. The strength of the repair was assessed at time zero, which eliminated the effect of long-term healing, graft remodelling and union. Lastly, all repairs used standard arthroscopic equipment but were not performed under surgical conditions.

This controlled laboratory study on sawbone models found that both the acromion and distal clavicle are suitable autologous bone graft options for treating large anterior glenoid defects, having appropriate dimensions and contours for reconstructing the glenoid arc. The modified buckle-down and cross-link techniques are two graft fixation techniques that restore stability to the shoulder joint upon repairing a large glenoid defect and are advantageous in being screw-free and simple to execute.

## Figures and Tables

**Table 1:** Intraclass correlation coefficients after testing 30 intact glenoids with the associated measurement.

Measurement	Intraclass Correlation Coefficients n = 30 (95% Confidence Interval)
2 mm Load	0.85 (0.62–0.95)
2 mm Contact Pressure	0.88 (0.68–0.96)
4 mm Load	0.81 (0.43–0.94)
4 mm Contact Pressure	0.88 (0.64–0.96)
Peak Translational Force	0.80 (0.41–0.93)
Maximum Contact Pressure	0.90 (0.72–0.96)

**Table 2:** Dimensions of acromion and clavicle grafts (mean ± SD).

Graft	Width (mm)	Length (mm)	Height (mm)
Acromion	11.5 ± 0.5	26.0 ± 0.4	11.5 ± 0.3
Clavicle	11.3 ± 0.5	27.7 ± 0.8	12.0 ± 0.6

## Data Availability

The data presented in this study are available on request from the corresponding author.
